# Brain Connectivity Dissociates Responsiveness from Drug Exposure during Propofol-Induced Transitions of Consciousness

**DOI:** 10.1371/journal.pcbi.1004669

**Published:** 2016-01-14

**Authors:** Srivas Chennu, Stuart O’Connor, Ram Adapa, David K. Menon, Tristan A. Bekinschtein

**Affiliations:** 1 Department of Clinical Neurosciences, University of Cambridge, Cambridge, United Kingdom; 2 Medical Research Council, Cognition and Brain Sciences Unit, Cambridge, United Kingdom; 3 Warwick Medical School, University of Warwick, Coventry, United Kingdom; 4 Division of Anaesthesia, University of Cambridge, Cambridge, United Kingdom; 5 Department of Psychology, University of Cambridge, Cambridge, United Kingdom; University of Pennsylvania, UNITED STATES

## Abstract

Accurately measuring the neural correlates of consciousness is a grand challenge for neuroscience. Despite theoretical advances, developing reliable brain measures to track the loss of reportable consciousness during sedation is hampered by significant individual variability in susceptibility to anaesthetics. We addressed this challenge using high-density electroencephalography to characterise changes in brain networks during propofol sedation. Assessments of spectral connectivity networks before, during and after sedation were combined with measurements of behavioural responsiveness and drug concentrations in blood. Strikingly, we found that participants who had weaker alpha band networks *at baseline* were more likely to become unresponsive during sedation, despite registering similar levels of drug in blood. In contrast, phase-amplitude coupling between slow and alpha oscillations correlated with drug concentrations in blood. Our findings highlight novel markers that prognosticate individual differences in susceptibility to propofol and track drug exposure. These advances could inform accurate drug titration and brain state monitoring during anaesthesia.

## Author Summary

Though scientific understanding of how brain networks generate consciousness has seen rapid advances in recent years, application of this knowledge to accurately track transitions to unconsciousness during general anaesthesia has proven difficult due to considerable variability in this gradual process across individuals. Using high-density electroencephalography, we studied changes in these networks as healthy adults were sedated using propofol. By measuring their behavioural responsiveness and amount of sedative in their blood, we found a striking pattern: the strength of their brain networks before sedation predicted why some participants lost consciousness while others did not, despite registering similar blood levels of drug. By uncovering underlying signatures of this variability, our findings could enable accurate brain monitoring during anaesthesia and minimise intra-operative awareness.

## Introduction

Understanding how the human brain reversibly generates and loses consciousness, through complex interactions of neural activity at multiple spatial and temporal scales, is a grand challenge for modern neuroscience. Recent theoretical advances have argued that consciousness changes when the balance between integrated and differentiated neural activity is affected [1–4]. However, accurately tracking these changes in brain dynamics remains a key research challenge with potentially wide-ranging applications, and is complicated by the significant individual variability in the trajectory along which consciousness is lost and regained.

The process of reversibly inducing unconsciousness using anaesthetic drugs like propofol is commonplace in clinical medicine [5]. However, tracking brain activity to accurately assess the depth of anaesthesia in an individual is currently not a universal component of clinical practice. Indeed, surface electroencephalography (EEG) is relatively easy to measure from the scalp and has long been known to index changes in brain dynamics induced by anaesthetic action [6], but it is still not universally used in the clinical setting. This is despite the fact that intraoperative awareness during surgery continues to result in pain and distress [7], highlighting the need for reliable depth of anaesthesia monitoring in the operating room. The absence of ubiquitous brain monitoring during general anaesthesia is, in part, due to the lack of robust EEG markers derived from current advances in neuroscience [8–12], which can accurately track the loss and reestablishment of reportable consciousness. Monitoring of brain states is currently limited to proprietary systems with mixed results [13–15]. Crucially, one reason for this is the considerable individual variability in susceptibility to anaesthetic dosage [16], which adversely affects the accuracy of these systems [17].

To better understand the factors underlying this variability, we combined the measurement of high-density resting state EEG from healthy volunteers sedated with propofol with measurement of drug concentrations in blood, in addition to objective assessment of behavioural responsiveness. With this aim in mind, we administered propofol at dosages expressly aimed at engendering varying degrees of mild to moderate sedation across our participant group, rather than complete unconsciousness in all of them. Employing modern functional EEG tools to assess spectral power and connectivity, we identified key changes in brain networks using graph-theoretic tools, and linked these changes to individual variability in drug concentrations and loss of behavioural acuity during sedation. Drawing upon previous research [18–21], we hypothesised characteristic impairments in the strength and topography of EEG power and connectivity, especially manifesting in the slow and alpha frequency bands, alongside administration of propofol. In addition to confirming these hypotheses, our findings highlight valuable EEG-derived signatures that can not only track the actual amount of propofol in blood, but also predict loss of responsiveness even before any drug is administered. These findings contribute to the current interest in identifying consistent markers of the loss and recovery of consciousness during propofol sedation. In the clinical context, these findings could lead to more accurate drug titration and brain state monitoring during anaesthesia.

## Results

### Drug and behaviour are distinct variables

**Fig 1:**
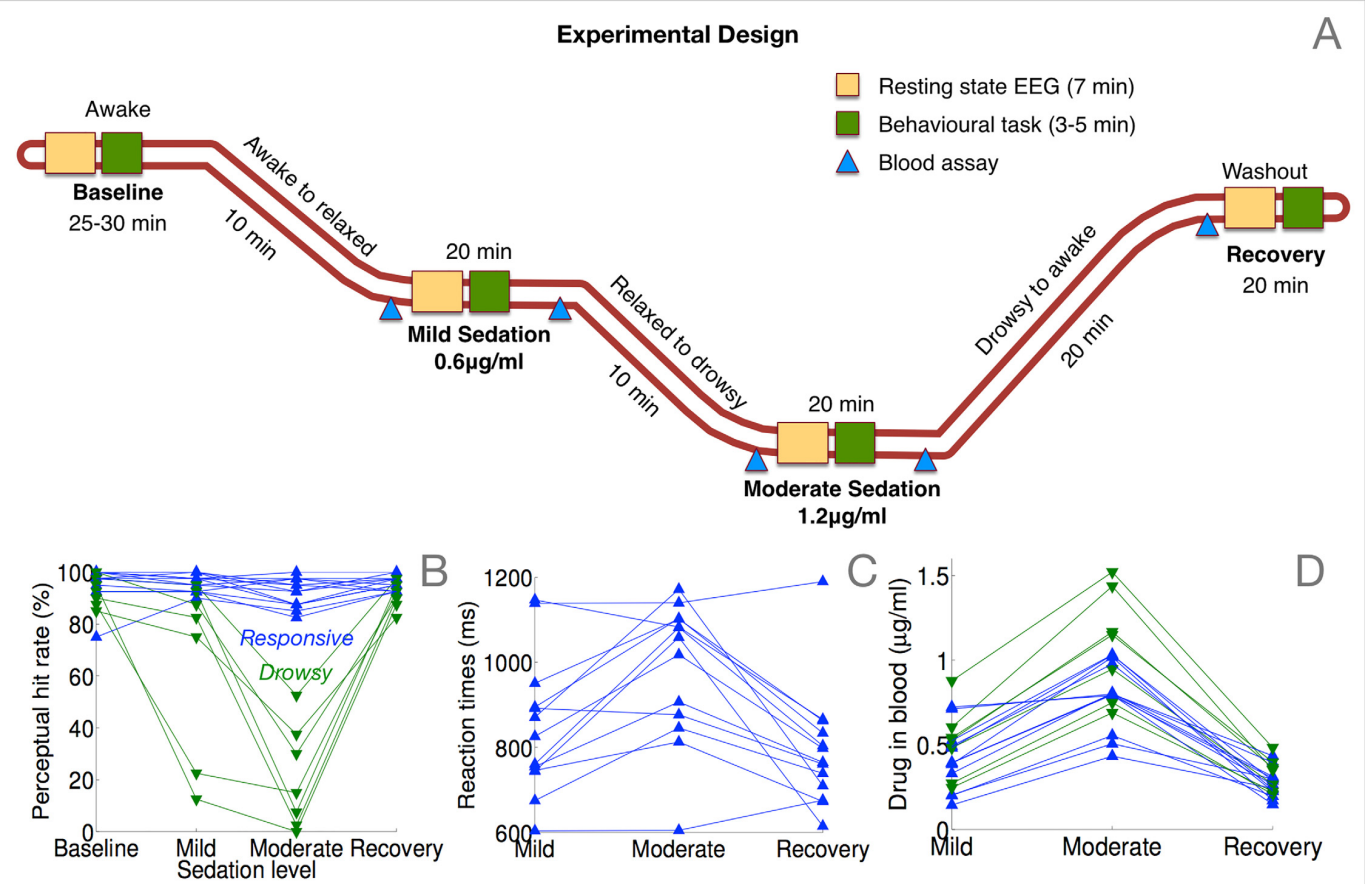
Experimental manipulation and measurement of behaviour and propofol concentration in blood plasma. (A) Resting state EEG data were collected for four ~7 minute periods from each participant: at baseline before administration of propofol, at mild sedation, moderate sedation, and finally at recovery. Each resting state data collection was followed by a two-choice speeded response task to assess behavioural responsiveness. Blood samples were collected and analysed offline to measure and correlate actual levels of propofol in plasma with EEG measures. (B) Two sub-groups of participants, *responsive* and *drowsy*, were identified based on binomial modelling of the change in their behavioural responsiveness due to sedation. (C) Reaction times in the responsive group were slower during moderate sedation. (D) Measured drug concentrations in blood plasma overlapped between the two groups.

The behavioural changes accompanying the administration of progressively increasing amounts of propofol (Fig 1A) are shown in Fig 1B, which plots the hit rate of participants as a function of the level of sedation. Based on binomial modelling of their hit rates (see Materials and Methods), we identified a subgroup of 7 participants who became behaviourally impaired at this simple task during moderate sedation; 13 others remained responsive throughout, though their reaction times were impaired during sedation (Fig 1C). We designate these two groups as *drowsy* (green triangles) and *responsive* (blue triangles) in the following descriptions. As expected, we found a highly significant interaction between group and sedation level in hit rates (Fig 1B; F(3) = 38.4, p = 9e-09). Further, in the responsive group, there was a significant effect of sedation on reaction times (Fig 1C; F(2) = 14.6, p = 0.0002).

In comparison to the relative distinction between the two groups in their hit rates, there was considerably more overlap in drug concentrations measured in blood plasma (Fig 1D). We found a relatively weaker interaction between group and level of sedation in drug concentrations: F(2) = 4.7, p = 0.0242, and the difference between drug concentrations in the two groups reached significance only during moderate sedation (p = 0.0181). This finding points to the well-studied inter-individual variability in pharmacodynamic impact of propofol [16, 17], and motivates the development of more accurate signatures of responsiveness that can be measured passively and non-invasively during propofol sedation.

### Alpha connectivity is compromised during sedation

**Fig 2:**
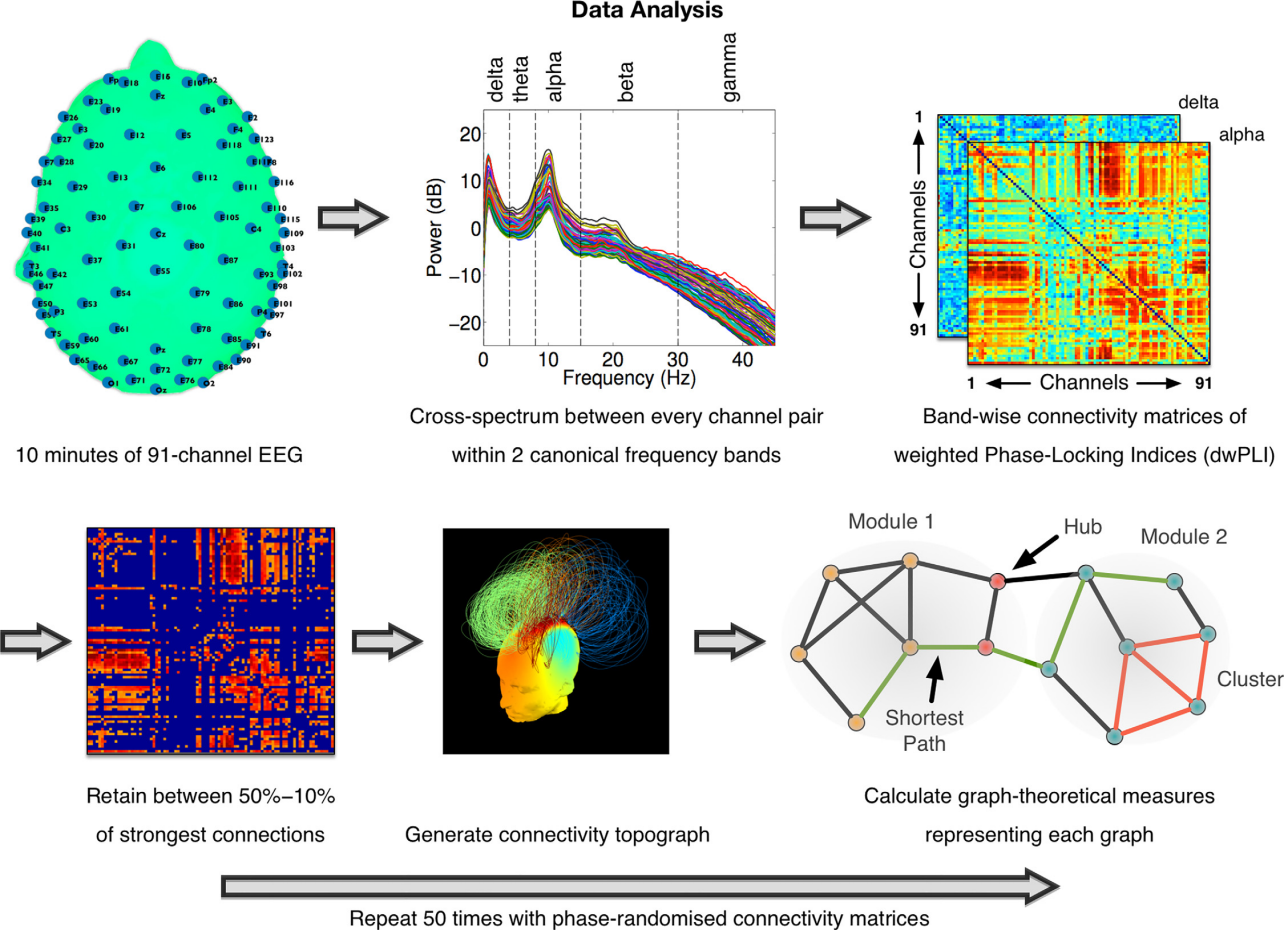
Summary of EEG data analysis pipeline. Cross-spectral density between channel pairs was estimated using dwPLI. Symmetric connectivity matrices generated were thresholded before the estimation of graph-theoretic metrics. In the connectivity matrix shown (bottom left), the threshold has been set to depict only top 30% of strongest connections. In the network topograph (bottom middle), intra-modular links in modules identified by the Louvain algorithm are indicated by colour.

Connectivity between EEG channels was assessed to directly investigate the impact of propofol on the structure of brain networks of oscillatory neural interactions, using the debiased weighted Phase Lag Index (dwPLI, see Fig 2 and [22]). Here, we define brain networks as the characteristic patterns of scalp-level connectivity observable in human EEG at different frequencies, generated by underlying cortical networks [23] with firing rates oscillating at their natural frequencies [24]. We employed the dwPLI connectivity matrices in each band to construct such EEG-derived brain networks, and used graph-theoretic algorithms to quantitatively compare their topological properties. By representing the EEG channels as nodes of a network and the strength of dwPLI between them as weighted, undirected links between them, we calculated four measures that captured micro-scale (*clustering coefficient*), meso-scale (*modularity* and *participation coefficient*) and macro-scale properties (*characteristic path length*) of each participant’s network at each level of sedation (see bottom right panel of Fig 2 for a visual description of these properties). Importantly, these metrics were chosen *a priori* to summarise key network properties that we expected to be modulated during propofol sedation.

**Fig 3:**
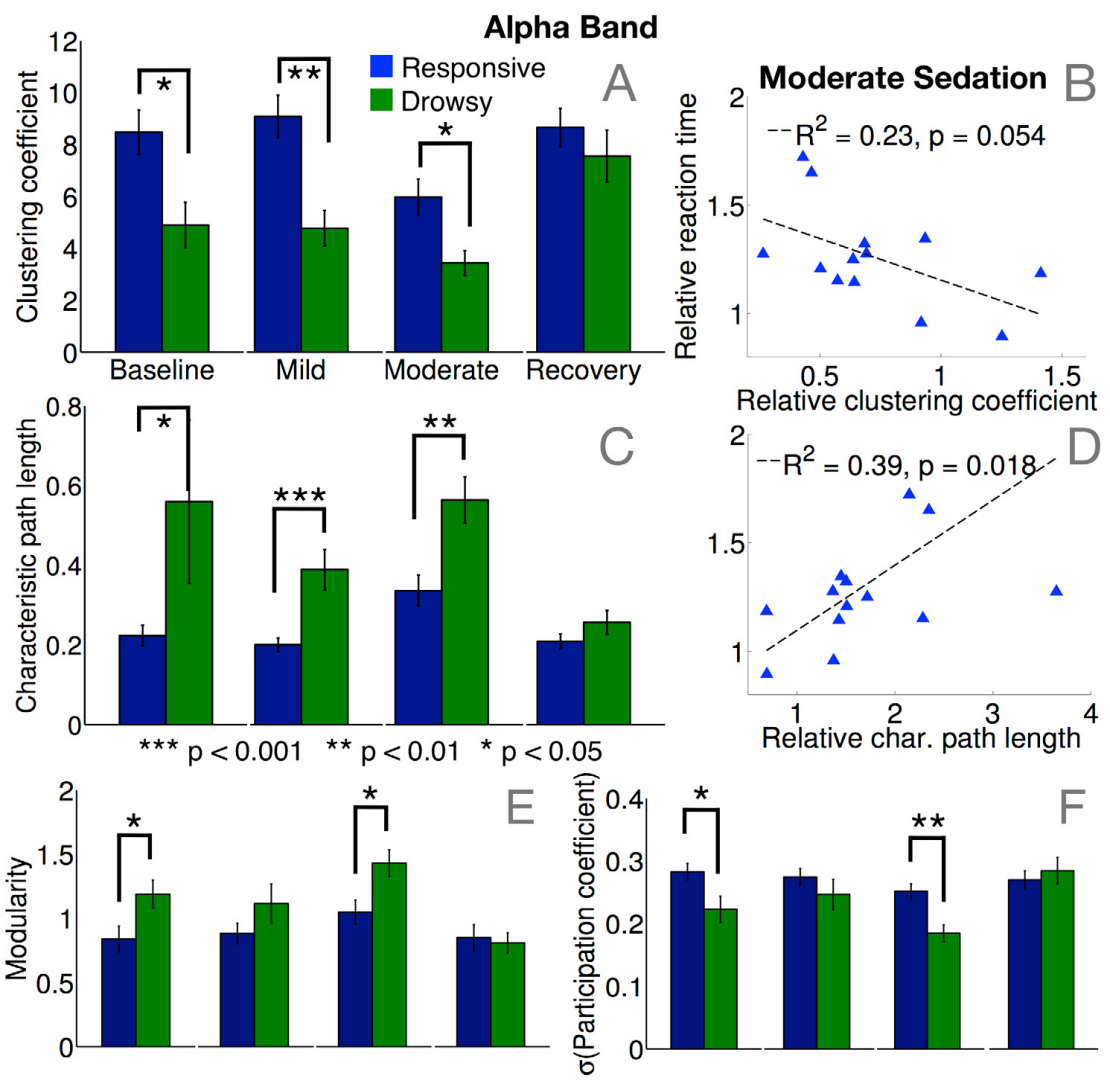
Summary of alpha connectivity changes. There were significant differences in the levels of local clustering (A) and global path lengths (C) between responsive and drowsy groups during mild and moderate sedation. Alpha networks in the drowsy group were hence significantly less locally and globally efficient. Crucially, these differences between the groups were apparent even at baseline, when the groups were behaviourally indistinguishable. Within the responsive group, decreasing levels of local (B) and global (D) efficiency were associated with slowing of reaction times during moderate sedation, relative to baseline. There were also significant differences in meso-scale modularity (E) and the presence of hub-like nodes with high participation coefficients (F) between responsive and drowsy groups during moderate sedation. Alpha networks in the drowsy group were more modular, with weaker hubs, even at baseline. Error bars depict standard error of the mean.

In the alpha band, median dwPLI across all channel pairs was significantly more reduced in the drowsy group during mild (p = 0.003) and moderate sedation (p = 0.01). Further, the clustering coefficient [25, 26], which measures local efficiency, was significantly lower (Fig 3A) in the frontal alpha networks of the drowsy group during mild (p = 0.007) and moderate sedation (p = 0.04). Furthermore, within the responsive group, clustering during moderate sedation tended to decrease linearly alongside increasing reaction times (Fig 3B), though this effect only approached significance. Conversely, characteristic path length (Fig 3C), the inverse of global efficiency, was significantly higher during mild (p = 0.0004) and moderate sedation (p = 0.0035), and tended to increase with slower reaction times among responsive participants (Fig 3D). Taken together, *small-worldness*, a combined measure of a network’s local and global efficiency (calculated as the ratio of clustering to path length [26, 27]), was significantly reduced in the drowsy group during mild (p = 0.005) and moderate sedation (p = 0.03). At the meso-scale, these drowsy alpha networks were also more modular at moderate sedation (Fig 3E, p = 0.02), and hence more separable into relatively disconnected topological modules [28]. Crucially, these modules lacked hub nodes that connected them into an integrated network, as evidenced by statistically lower standard deviation (p = 0.002) of participation coefficients [29] in the drowsy group (Fig 3F). Together, these network differences demonstrated that the frontal alpha connectivity in the drowsy group did not have the network capacity of the occipital alpha network commonly observed in human resting EEG during wakefulness.

**Fig 4:**
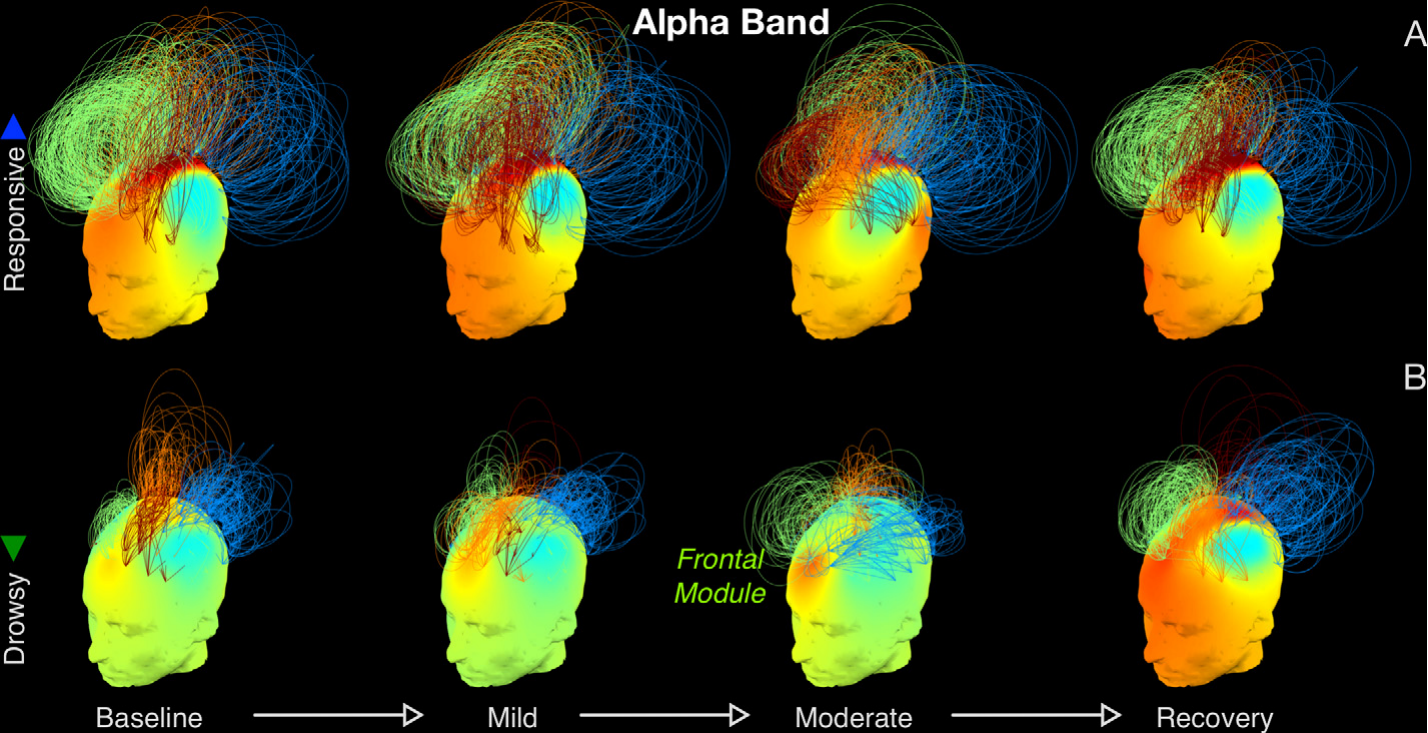
Alpha band connectivity networks. Network topographs are visualised at each level of sedation, averaged over participants in the responsive (A) and drowsy (B) groups. Colour of the scalp indicates the degree of a node. The normalised height of an arc connecting two nodes indicates the strength of the dwPLI link between them. Network modules were identified by the Louvain algorithm. For visual clarity, of the strongest 30% of links, only the intra-modular links are plotted. The colour of an arc identifies the module to which it belongs. While alpha band networks in the responsive group exhibited a relatively stable fronto-centro-occipital pattern of connectivity, the drowsy group altered significantly, and registered a pronounced frontally centered module during moderate sedation.

**Fig 5:**
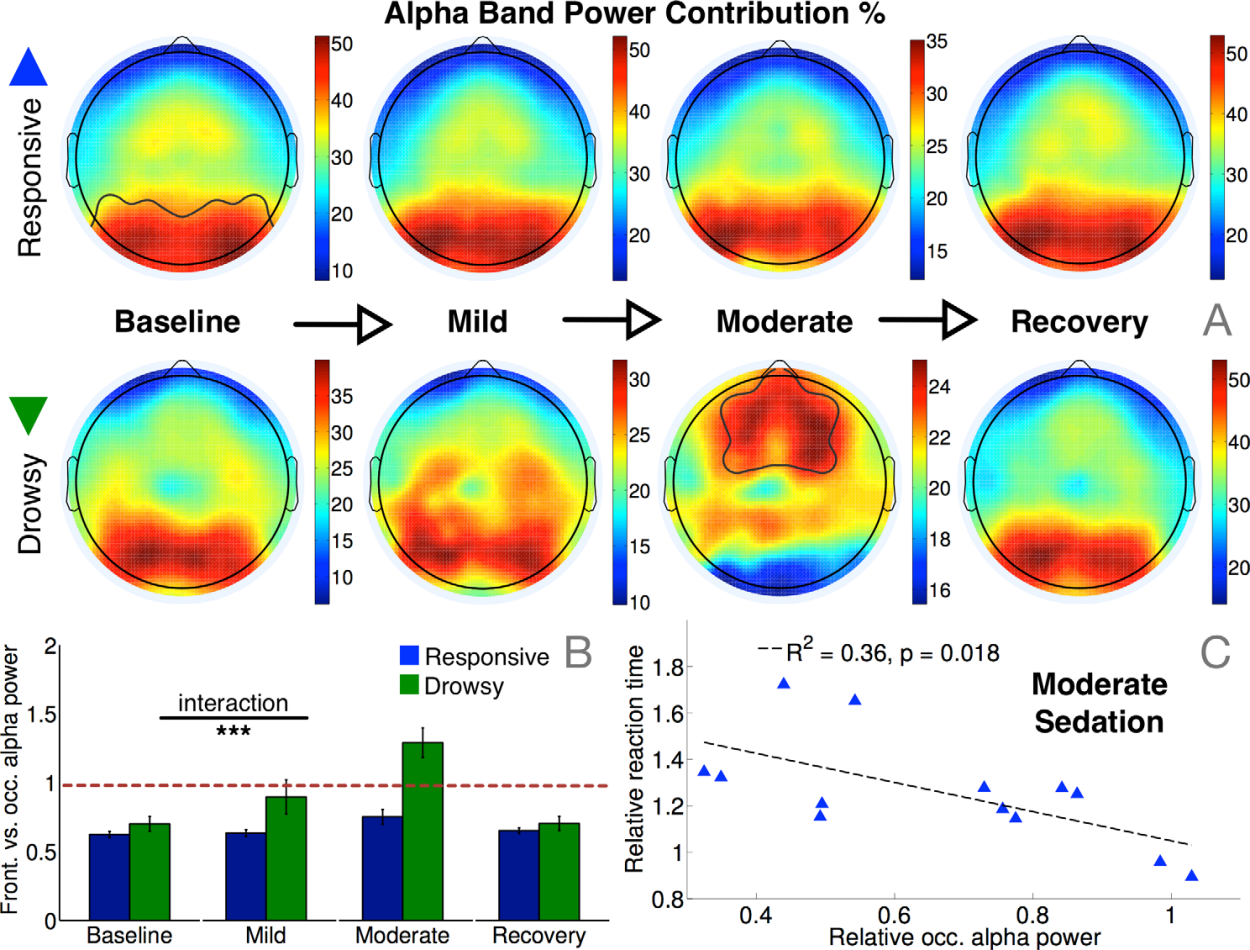
Alpha band power changes as a function of sedation. (A) Alpha power topography in the drowsy group progressively switched from an occipital to a frontal pattern during moderate sedation, while the responsive group remained stable throughout. (B) The resulting interaction between group and level of sedation on their alpha power contributions from frontal vs. occipital channels was statistically significant (F(3) = 10.1, p = 0.0008). (C) Even amongst the responsive group, reduction in relative occipital alpha power during moderate sedation was correlated with relatively slower reaction time, relative to baseline.

These changes in alpha networks can be understood more visually with Fig 4A. At baseline, both groups had prominent frontocentral and occipital modules of strong connectivity. While these modules persisted through moderate sedation in the responsive group, the structure of connectivity networks in the drowsy group shifted to qualitatively distinct state comprising of coherent, frontally centered oscillations that manifested as a frontal module (Fig 4B), before reverting back to the typical pattern of baseline connectivity during recovery. On the whole, this shift in alpha connectivity mirrors the frontal shift in alpha power (Fig 5) commonly observed during propofol sedation [18, 19, 30–32]. In contrast to these changes in alpha networks, no differences were observed between delta networks in the two groups (see S1 Fig).

### Alpha network properties before sedation predict susceptibility to propofol

Spectral connectivity in the alpha band identified a prospectively valuable determinant of the variability in susceptibility to propofol seen in the behavioural data. During the baseline period before sedation, though there were no differences in the topography or relative strength of alpha power between the responsive and drowsy groups (Fig 5A and 5B), there were significant differences in median dwPLI (p = 0.0085) and key network properties that captured the topological structure of connectivity in the alpha band. Specifically, alpha networks in the drowsy group were already less clustered (Fig 3A; p = 0.04) and less small-worldy (p = 0.0187) at baseline. They were also more modular (Fig 3E; p = 0.04), and had fewer hubs (Fig 3F; p = 0.0018). Remarkably, these baseline alpha network differences were evident when the two groups of participants were indistinguishable, both in terms of behavioural hit rates (Fig 1B) and occipital alpha power (Fig 5B). Furthermore, this predictive value of brain connectivity was unique and specific to the alpha band, and not evident in other frequency bands (see S1 Fig). In line with previous findings [31, 33, 34], sedation selectively increased beta/gamma power and connectivity among responsive participants, but baseline power or connectivity in these bands was not significantly different between the two groups.

**Fig 6:**
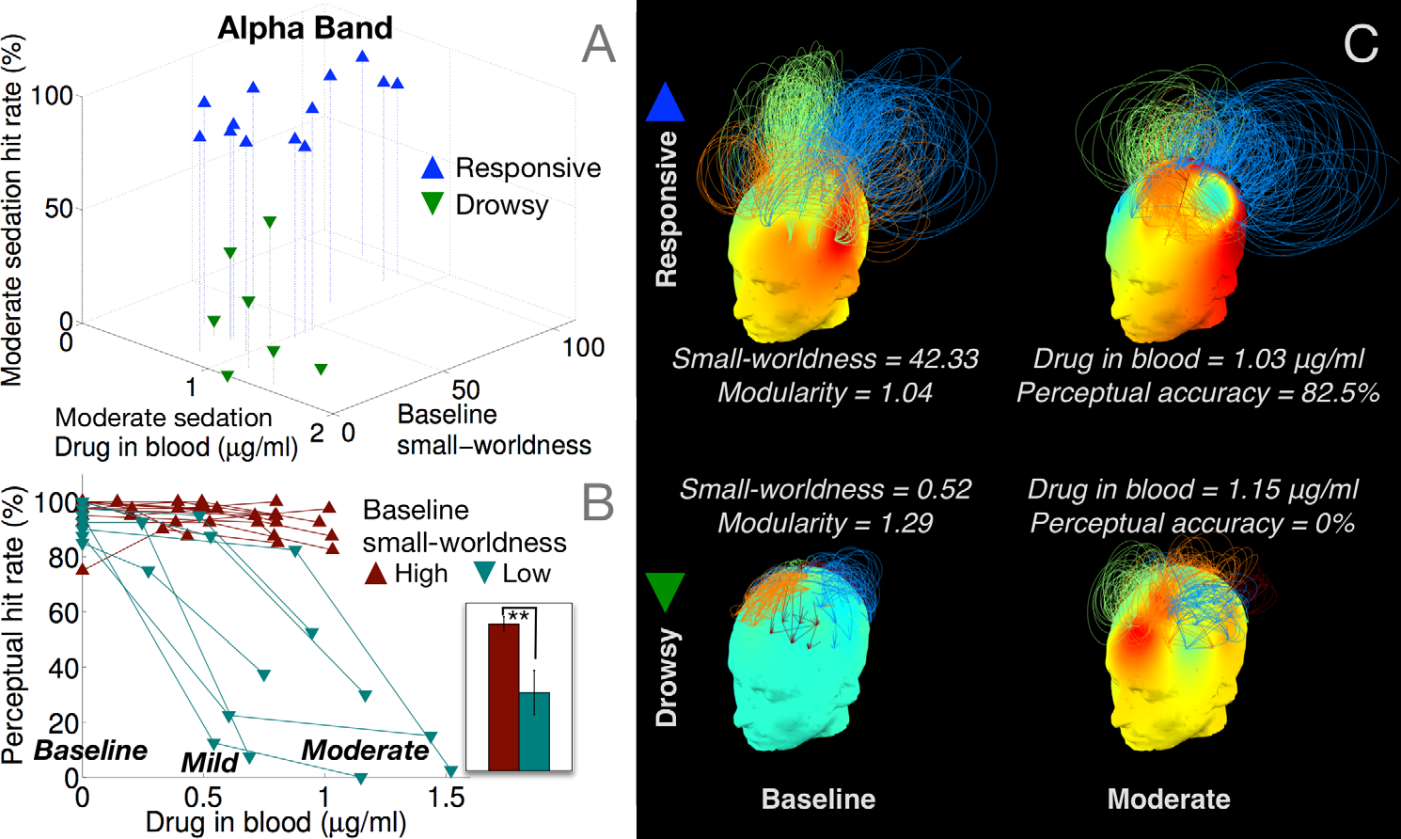
Baseline alpha band networks predict loss of responsiveness during moderate sedation. Participants in the drowsy group had relatively lower small-worldness (A) already at baseline. Median split of participants based on baseline small-worldness predicted eventual loss of responsiveness (B) despite similar blood levels of drug concentration during moderate sedation (C).

To explicate this result further, Fig 6A depicts a scatter plot of alpha network small-worldness in each participant measured during pre-drug baseline, against their consequent behavioural hit rates and drug concentrations measured during moderate sedation. Though there was considerable variability in small-worldness across the responsive group at baseline, the drowsy group already had relatively lower small-worldness in comparison. To directly test whether participants who already had less robust brain networks at baseline later became drowsy or unresponsive during moderate sedation, Fig 6B plots the individual hit rate trajectories of the participants separated based on whether their baseline small-worldness was above or below the median. Those in the group with high baseline small-worldness remained responsive, and had significantly higher hit rates during moderate sedation (Fig 6B, inset; p = 0.0093). This predictive role of alpha brain networks in characterising individual variability in susceptibility to propofol is exemplified in Fig 6C, which depicts their evolution in two ‘drug concentration-matched’ participants. Despite registering relatively similar drug concentrations at moderate sedation, one of them remained responsive while the other became completely unresponsive. As is evident, the latter participant already had a comparatively less robust alpha network already at baseline, which then evolved into a frontally alpha module at moderate sedation. In comparison, the responsive participant had a relatively more small-worldy, less modular network at baseline, which was sustained during moderate sedation. These differences potentially explain why the drowsy group, whose alpha networks were already compromised to some degree, became behaviourally impaired while the responsive group did not, despite both groups registering overlapping levels of propofol as measured in their blood at moderate sedation. It is important to note that these differences observed in the baseline alpha networks were abolished at recovery (see Fig 3A and 3C). This suggested that these differences between the two groups were essentially dependent on the latent alpha network state of the participants at the beginning of the data collection rather than any individual trait, and were ‘reset’ after the washout of the drug.

### Slow-alpha phase-amplitude coupling is linked to drug concentrations in blood

**Fig 7:**
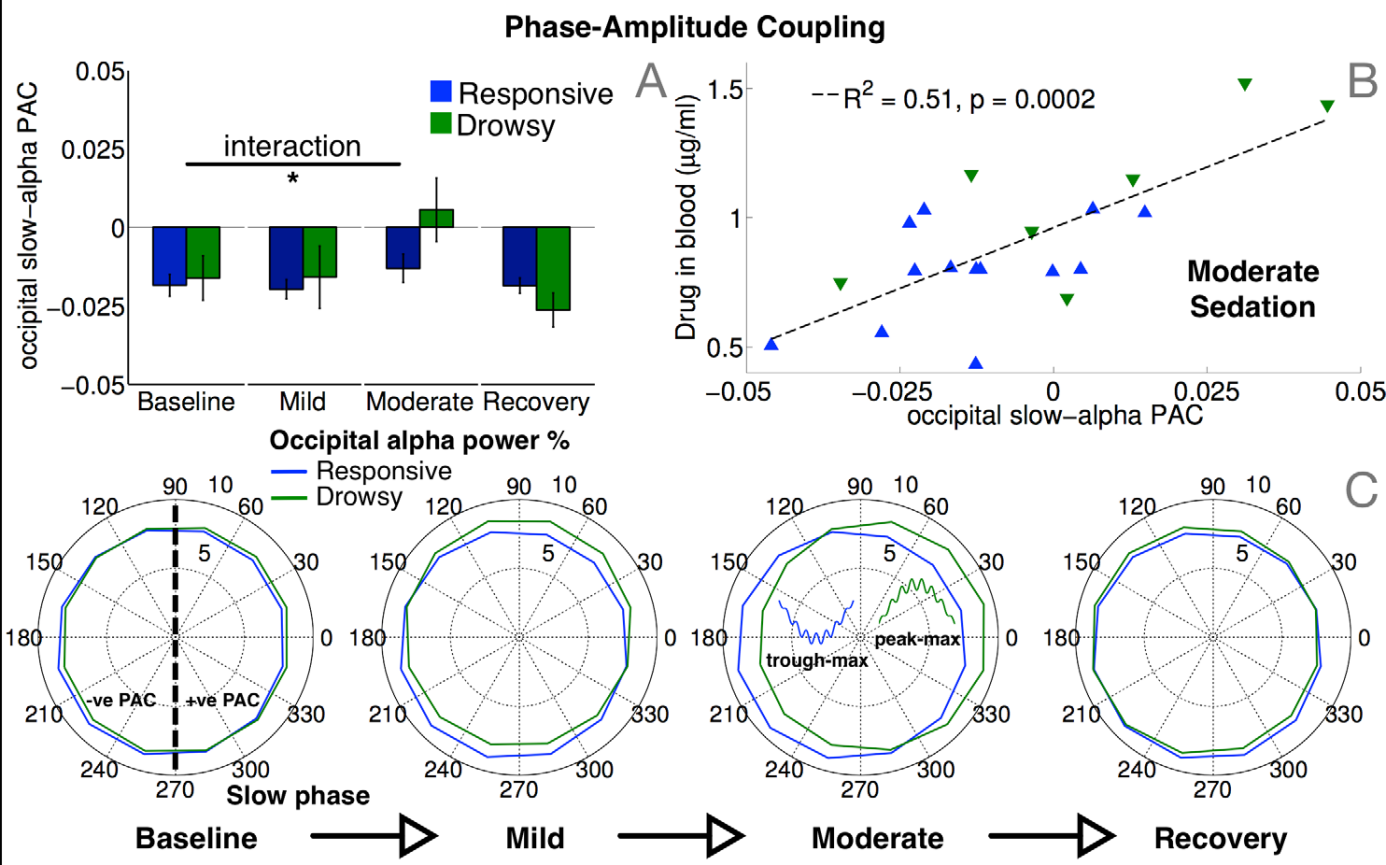
Phase-amplitude coupling (PAC) between slow and alpha oscillations. Coupling between ongoing slow phase and alpha power over occipital channels delineated in Fig 5A (top left) shifted from a trough-max to a peak-max (C) distribution in the drowsy group during moderate sedation, resulting in a significant interaction between group and sedation in PAC values (A). Crucially, these subject-wise PAC values significantly correlated with drug concentrations measured in blood across both groups during moderate sedation (B).

We found that, at baseline, participants in both responsive and drowsy groups had similar temporal coupling between the phase of slow oscillations and alpha power, with negative values of phase-amplitude coupling (PAC; Fig 7A) over occipital channels (delineated in Fig 5A, top left). This pattern persisted during mild sedation and only changed during moderate sedation within the drowsy group, in whom it shifted toward positive PAC values, before reverting back to negative PAC at recovery. There was a significant interaction in occipital PAC between level of sedation and group (F(3) = 3.8, p = 0.021). Fig 7C provides more detail on this, using angular histograms of alpha power distributed over slow phase, for a pair of representative participants, one in each of the two groups, responsive and drowsy. At baseline, occipital alpha power was either evenly spread over slow phase, or was greater near the trough of the slow oscillation, resulting in a trough-max distribution and negative PAC. During moderate sedation, only the drowsy participant’s distribution shifted towards peak-max positive PAC with greater alpha power near slow oscillation peaks. At recovery, this distribution reverted back to a trough-max pattern with negative PAC. Further, we also found a highly significant positive correlation between PAC and drug concentrations in blood during moderate sedation (Fig 7B). This correlation did not manifest during mild sedation or recovery, when drug concentrations were relatively low. Importantly, there was no significant correlation between PAC and reaction times. This was in contrast to the correlations between alpha power/connectivity and reaction times (Figs 3B and 3D and 5C), and highlights a novel dissociation between phase-phase and phase-amplitude coupling: while the former correlated with responsiveness as measured by hit rates and reaction times, the latter correlated drug concentrations in blood.

Juxtaposed with previous research, our findings are convergent with existing evidence for characteristic changes in PAC alongside propofol induction. Trough-max slow-alpha PAC has been shown to accompany transitions to unconsciousness in frontal EEG channels, which then switches to a peak-max pattern in the same channels following loss of consciousness during deep sedation [18, 35]. While we have highlighted complementary changes in occipital channels, we also replicated these previous findings. In frontal channels, slow-alpha PAC values were close to zero at baseline, and progressed to a trough-max pattern during moderate sedation (see S2 Fig). This resulted in a significant interaction between level of sedation and group in frontal PAC values (F(3) = 4.1, p = 0.0136), with the drowsy group showing a significantly stronger trough-max pattern than the responsive group during moderate sedation (p = 0.011). Further, as with occipital PAC, there was a significant correlation between frontal PAC and drug concentrations in blood during moderate sedation (S2 Fig).

## Discussion

Our experimental design used propofol sedation to engender transitional states of responsiveness that varied across participants. The levels of drug administered produced a variable pattern that spread the participant group along a spectrum of varying behavioural impairment, rather than resulting in complete unconsciousness in all of them. Using EEG to track brain activity and measuring actual levels of drug in blood alongside this spectrum of impairment has enabled us to identify neural markers that dissociate conscious report from drug exposure [2], and makes the results presented here distinctive in their contribution to advancing understanding of the neural markers of loss of consciousness due to propofol.

We have built upon previous research that has shown that while occipital alpha power progressively drops as participants become behaviourally compromised as measured by reaction times, the qualitatively dissimilar onset of frontal alpha power is a characteristic marker of the loss of consciousness [18, 19, 30, 32, 36]. Confirming our hypotheses, while this frontal alpha generates meso-synchronous modules, brain network connectivity as a whole is nevertheless impaired. Graph-theoretic measures quantify this loss of the capacity of individual brain networks in the alpha band, linking them to concomitant variability in behavioural impairment across participants. Small-worldness is commonly seen as a measure of the cost-versus-efficiency optimality of a network configuration, and our findings converge with previous evidence [37] highlighting the reduction in the efficiency of cortical networks during loss of consciousness during propofol sedation, potentially due to dysfunctional modulations in thalamocortical connectivity [8, 38, 39]. It is worth noting that a similar breakdown in the capacity of alpha networks has also been reported with other anaesthetic agents like sevoflurane and ketamine [40–42]. This is despite the fact that these distinct anaesthetic agents had varying effects on EEG oscillations and, unlike propofol, did not always produce increases in frontal alpha. Hence the observed changes in alpha networks due to sedation cannot be explained as a shift of alpha power and connectivity from posterior to anterior areas. Rather, our results, along with these previous findings, point toward a broader understanding of characteristic signatures of connectivity in alpha networks as potentially reliable correlates of reportable consciousness [43].

Measurement of drug concentrations at each level of sedation dissociated a principal clinical pharmacodynamics target per se (sedation and consequent behavioural unresponsiveness) from incidental pharmacodynamic consequences of drug exposure during propofol sedation. The considerable individual variability in the susceptibility to anaesthesia has been documented [16], and is evident in the large overlap between blood levels of drug in our responsive and drowsy groups. While our measurement of modulations in phase-phase coupling in delta and alpha bands during sedation showed clear correlations with behavioural impairment, we have also demonstrated a latent relationship between slow-alpha phase coupling and individual variation in drug concentrations. It is important to distinguish these dynamic slow oscillations from stable slow cortical potentials observed during propofol anaesthesia [12], and from delta oscillations during sleep [44]. This link between PAC and individual levels of drug in blood was not observed in the delta or alpha bands separately, in either power or connectivity. Analytical approaches used for estimating Bispectral Index (BIS, see [45] that do not take phase information into account are unlikely to detect this key marker of individual drug concentration [18]. Hence our findings are relevant to the challenge of engendering an appropriate level of unconsciousness by accurately tailoring drug concentrations to individuals, a key consideration with significant implications for clinical anaesthesia.

Finally, by tracking individual brain networks across levels of sedation, we have shown that the quantifiable robustness of alpha connectivity networks in the awake state *before* sedation predicts susceptibility to propofol. Specifically, given two behaviourally indistinguishable individuals undergoing administration of sedative, the one with the more robust, small-worldy alpha network with well-connected hubs is likely to require a greater amount of drug to render them unresponsive to the same degree. It is important to note that this *latent* variability in the state of alpha connectivity at baseline could be detected despite the lack of any significant differences in behavioural performance or alpha power at that time. Orthogonally, slow-alpha PAC complements this predictive capability by tracking the concentration of propofol in blood plasma. This set of results, if replicated and verified in the clinical context, could contribute to reliable applications of brain monitoring for tracking and accurately modulating consciousness with anaesthetics during routine surgery.

## Materials and Methods

### Ethics statement

All healthy controls gave written informed consent. Ethical approval for testing healthy controls was provided by the Cambridgeshire 2 Regional Ethics Committee. All clinical investigations were conducted in accordance with the *Declaration of Helsinki*.

### Participants

A convenience sample of 22 neurologically healthy adults participated in the study. Data from two participants could not be used due to technical issues, leaving 20 participants (9 male; 11 female) (mean age = 30.85; SD = 10.98) whose data were analysed.

### Experimental protocol

Each experimental run began with an awake baseline period lasting 25–30 minutes (Fig 1A) following which a target-controlled infusion of propofol [46] was commenced via a computerized syringe driver (Alaris Asena PK, Carefusion, Berkshire, UK). With such a system the anesthesiologist inputs the desired (“target”) plasma concentration, and the system then determines the required infusion rates to achieve and maintain the target concentration (using the patient characteristics which are covariates of the pharmacokinetic model). The Marsh model is routinely used in clinical practice to control propofol infusions for general anesthesia and for sedation.

Three blood plasma levels were targeted– 0.6μg/ml (mild sedation), 1.2μg/ml (moderate sedation), and recovery from sedation. The state of mild sedation was aimed to engender a relaxed but still responsive behavioural state. At each target level, a period of 10 minutes was allowed for equilibration of plasma propofol concentrations to attain a steady state, following which behavioural tests and EEG measurements were commenced. After cessation of infusion, plasma propofol concentration exponentially declined toward zero. Computer simulations with the *TIVATrainer* pharmacokinetic simulation software revealed that plasma concentration of propofol would approach zero in 15 minutes leading to behavioural recovery; hence behavioural assessment was recommenced 20 minutes after cessation of sedation. Blood samples of 1cc each were taken at the beginning and end of the mild and moderate sedation states, and once at recovery, as indicated in Fig 1A. In total, 5 blood samples were taken during the study. These samples were analysed offline for characterising the significant inter-individual variability in actual propofol levels in blood plasma. We confirmed that the samples taken at the beginning and end of mild and moderate sedation had similar values of propofol concentration. The average of the two values, along with the value at recovery, were used as distinct covariates for EEG data analysis.

### Behavioural data collection

At each of the 4 steady-state levels above, participants were requested to perform a simple behavioural task involving a fast discrimination between two possible auditory stimuli (Fig 1A). Specifically they were asked to respond with a button press to indicate whether a binaurally presented stimulus was a buzz or a noise. These stimuli constituted either broadband noise or a harmonic complex with a 150Hz fundamental frequency (buzz). Forty such stimuli, twenty of each kind, were presented in random order over two blocks, with a mean inter-stimulus interval of 3 seconds. We calculated a participant’s cognitive processing of these stimuli at each sedation level based on their *hit rates*, i.e., percentage of correct responses. In addition, we measured *reaction times* based on the delay between auditory tone onset and correct button press.

### Behavioural data analysis

We employed binomial modelling to distinguish participants who became behaviourally impaired during moderate sedation, from those who remained responsive, albeit with slower reaction times. Specifically, we fitted a binomial distribution to each participant’s hit rates at baseline and during moderate sedation. With each fitted model, the distribution parameter *p*, the probability of a correct response, and its 95% confidence intervals were estimated. For a given participant, if the confidence interval at moderate sedation was lower than and non-overlapping with that at baseline, they were considered to have become significantly impaired, and we designated them as *drowsy*. If the confidence intervals overlapped, we designated them as *responsive*.

### EEG data collection and pre-processing

From each participant, approximately 7 minutes of 128-channel high-density EEG data were collected at each level of sedation. EEG was measured in microvolts (uV), sampled at 250Hz and referenced to the vertex, using the Net Amps 300 amplifier (Electrical Geodesics Inc., Eugene, Oregon, USA). Participants had their eyes closed in a resting state during data collection.

Data from 91 channels over the scalp surface (Fig 2) were retained for further analysis. Channels on the neck, cheeks and forehead, which tended to contribute most of the movement-related noise, were excluded. Retained channels were filtered between 0.5–45Hz, and segmented into 10-second long epochs. Each epoch thus generated was baseline-corrected relative to the mean voltage over the entire epoch. Data containing excessive eye movement or muscular artefact were rejected by a quasi-automated procedure: abnormally noisy channels and epochs were identified by calculating their normalised variance and then manually rejected or retained by visual inspection. After pre-processing, a mean (SD) of 38 (5), 39 (4), 38 (4) and 40 (2) epochs were retained for further analysis in the baseline, mild sedation, moderate sedation and recovery conditions, respectively. An ANOVA revealed no statistically significant difference between the numbers of epochs retained. Finally, previously rejected channels were interpolated using spherical spline interpolation, and data were re-referenced to the average of all channels. These processing steps were implemented using custom MATLAB scripts based on EEGLAB [47].

### Spectral Power and connectivity analysis

Fig 2 depicts the data processing pipeline employed to calculate spectral power and connectivity measures from the clean EEG datasets. Spectral power values within bins of 0.25Hz were calculated using Fourier decomposition of data epochs using the *pwelch* method. At each channel, power values within canonical frequency bands, namely *delta* (0–4Hz), *theta* (4–8Hz), *alpha* (8–15Hz), *beta* (12-25Hz) and *gamma* (25–40Hz), were converted to relative percentage contributions to the total power over all five bands. Alongside, cross-spectrum between the time-frequency decompositions (at frequency bins of 0.49Hz and time bins of 0.04s) of every pair of channels was used to calculate debiased weighted Phase Lag Index (dwPLI, see [22]). For a particular channel pair and frequency band, mean dwPLI across all time at the peak frequency within each band was recorded as the ambient amount of connectivity between those channels. dwPLI is a sensitive measure of connectivity between cortical regions that has been shown to be robust against the influence of volume conduction, uncorrelated noise, and inter-subject variations in sample size [22], and has previously be used to characterise connectivity in pathological [48] and pharmacological [49] alterations in consciousness. However, as pointed out by Vinck, Oostenveld [22], dwPLI is relatively insensitive to true connectivity at phase differences close to 0 or 180 degrees. Further, the actual locations of brain sources producing dwPLI connectivity between a pair of sensors might not necessarily be spatially proximal to those sensors. Nevertheless, for the purposes of this study, it provides a robust measure for estimating how this indirect connectivity is affected by propofol sedation.

### Phase-amplitude coupling analysis

Phase-amplitude coupling (PAC), also referred to as cross-frequency coupling [50], was used to measure the propofol-induced changes in the relationship between the phase of ongoing oscillations in the slow (0.5–1.5Hz) and alpha (8–15Hz) bands at each channel. Calculation of PAC was based on the *Direct PAC* estimator formally defined by Ozkurt and Schnitzler [51] and implemented in the Brainstorm 3.2 toolbox [52]. Purdon, Pierce [18] and Mukamel, Pirondini [35] previously identified changes from *trough-max* to *peak-max* PAC during propofol sedation, as determined by whether the slow oscillation is at its trough (at a phase angle of *pi*) or its peak (phase angle of 0) when alpha power is maximal, respectively. Such variations were measured by assigning a *negative* or *positive* sign to the amplitude of the complex-valued *Direct PAC* estimator depending on whether its phase angle was closer to *pi* or *0* radians, to indicate *trough-max* and *peak-max* coupling respectively.

### Graph-theoretic analysis

The 91x91 subject-wise, band-wise dwPLI connectivity matrices were thresholded to retain between 50–10% of the largest dwPLI values. They were then represented as graphs with the channels as nodes and non-zero values as links between nodes. The lowest threshold of 10% ensured that the average degree was not smaller than 2 * log(*N*), where *N* is the number of nodes in the network (i.e., *N* = 91). This lower boundary guaranteed that the resulting networks could be estimated [26]. Similar ranges of graph *connection densities* have been shown to be the most sensitive to the estimation of ‘true’ topological structure therein [53, 54]: higher levels of connection density result in increasingly random graphs, while lower levels result in increasingly fragmented graphs.

At each step of the connection density between 50% and 10% in steps of 2.5%, the thresholded graphs were submitted to graph-theoretical algorithms implemented in the Brain Connectivity Toolbox [55]. These algorithms were employed to calculate metrics that captured key topological characteristics of the graphs at multiple scales, and avoided the multiple comparisons problem entailed by comparing large numbers of network connections. These included the micro-scale clustering coefficient and macro-scale characteristic path length [26], alongside meso-scale measures like modularity and community structure [56], and participation coefficient [29]. Here, this functional notion of modularity measures the extent to which the nodes of a graph can be parcellated into topologically distinct modules with more intra-modular links than inter-modular links [28]. Modularity as calculated by the heuristic Louvain algorithm, and all measures derived therefrom, were averaged over 50 repetitions. Next, each graph metric thus derived was normalised by the average of 50 null versions of the metric similarly derived, but after repeatedly phase-randomising the original cross-spectra and recalculating dwPLI for each channel pair. Finally, the small-worldness index of a graph was calculated as the ratio of normalised clustering coefficient to characteristic path length [57].

Metrics were compared using two-way ANOVAs with one non-repeated (group) measure and one repeated (level of sedation) measure. The obtained p-values were corrected for violations of sphericity using a Greenhouse-Geisser correction. Pairwise tests between groups were corrected for multiple comparisons using Tukey’s HSD test. The ability of graph metrics of individual participants to predict their behaviour was tested using robust linear regression [58] to calculate R^2^ and p-values.

## Supporting Information

S1 Fig Delta, theta, beta and gamma band power and connectivity as a function of sedation. (A) Delta power topography in the drowsy group showed prominent increases over occipital channels during moderate sedation, but there were no group differences in connectivity (B). Theta power decreased in both groups with sedation (C), with no differences in connectivity (D). In contrast, beta and gamma band power over bilateral frontocentral channels (E and G) and small-worldness (F and H) increased in the responsive but not the drowsy group. However, unlike in the alpha band, neither baseline power nor connectivity in any of these bands predicted later loss of responsiveness during moderate sedation.
(TIFF)

S2 Fig Phase-amplitude coupling (PAC) between slow and alpha bands in frontal channels. Coupling between ongoing slow phase and alpha power over frontal channels delineated in Fig 5A (bottom right) shifted from near zero to a trough-max distribution during moderate sedation, resulting in a significant interaction between group and sedation in PAC values (A). As seen in occipital channels, subject-wise PAC values in frontal channels significantly correlated with drug concentrations measured in blood across both groups during moderate sedation (B).
(TIFF)
