# Supplementary figures and images for: Brain Connectivity Dissociates Responsiveness from Drug Exposure during Propofol-Induced Transitions of Consciousness
Source: PLoS Comput Biol. 2016 Jan 14;12(1):e1004669. doi: 10.1371/journal.pcbi.1004669 (PMC4713143; doi:10.1371/journal.pcbi.1004669)

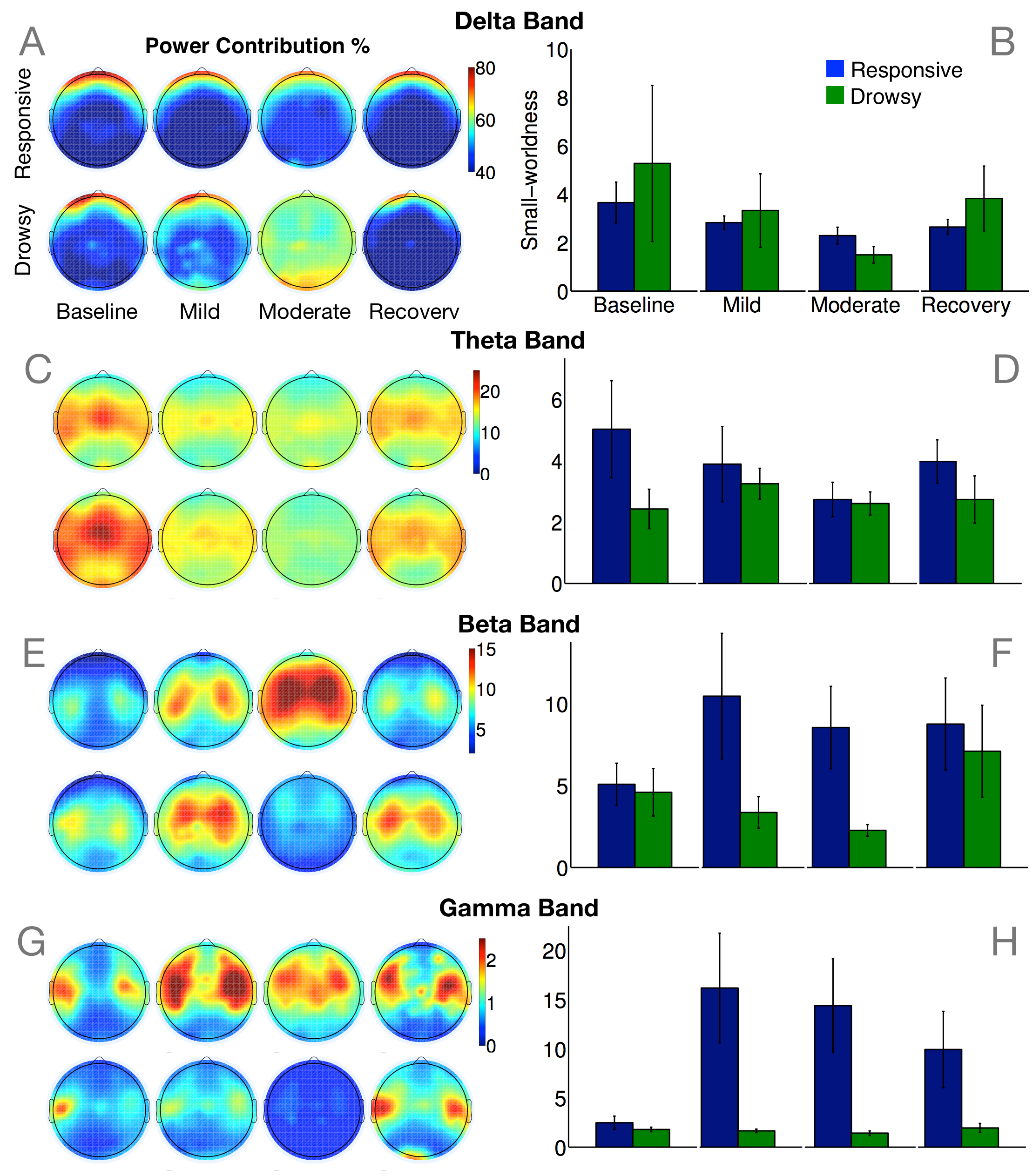

Supplement: S1 Fig — (A) Delta power topography in the drowsy group showed prominent increases over occipital channels during moderate sedation, but there were no group differences in connectivity (B). Theta power decreased in both groups with sedation (C), with no differences in connectivity (D). In contrast, beta and gamma band power over bilateral frontocentral channels (E and G) and small-worldness (F and H) increased in the responsive but not the drowsy group. However, unlike in the alpha band, neither baseline power nor connectivity in any of these bands predicted later loss of responsiveness during moderate sedation. (TIFF) [file pcbi.1004669.s001.tiff]

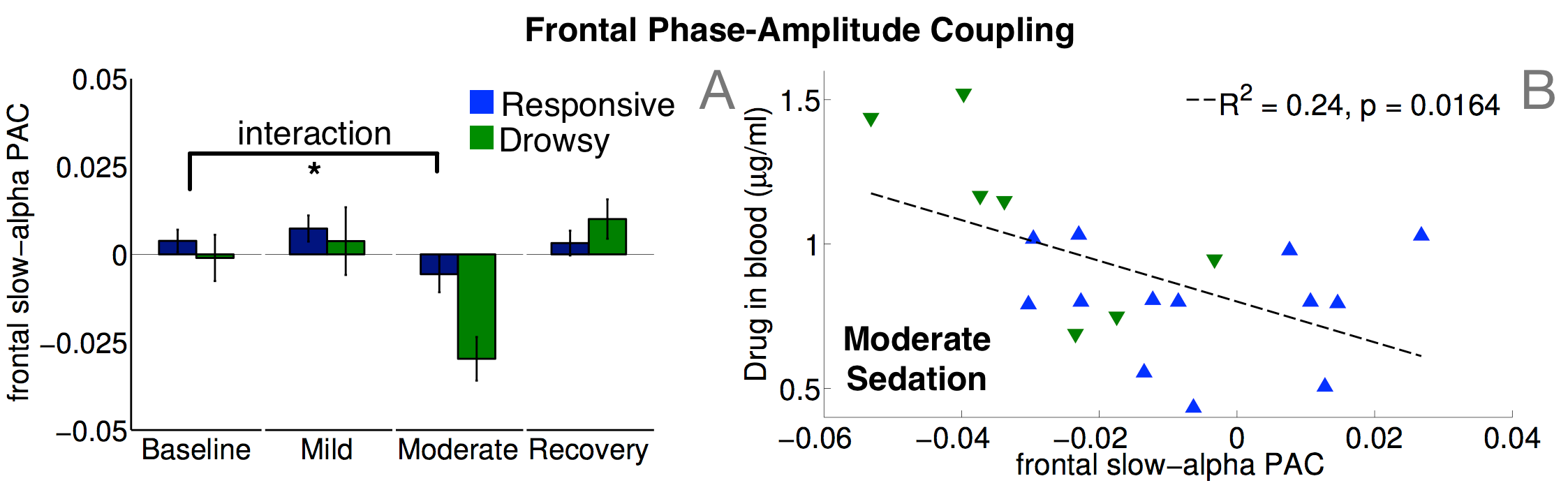

Supplement: S2 Fig — Coupling between ongoing slow phase and alpha power over frontal channels delineated in Fig 5A (bottom right) shifted from near zero to a trough-max distribution during moderate sedation, resulting in a significant interaction between group and sedation in PAC values (A). As seen in occipital channels, subject-wise PAC values in frontal channels significantly correlated with drug concentrations measured in blood across both groups during moderate sedation (B). (TIFF) [file pcbi.1004669.s002.tiff]
